# Nicotinamide N-Methyltransferase: A Promising Biomarker and Target for Human Cancer Therapy

**DOI:** 10.3389/fonc.2022.894744

**Published:** 2022-06-09

**Authors:** Xiao-Yu Li, Ya-Nan Pi, Yao Chen, Qi Zhu, Bai-Rong Xia

**Affiliations:** ^1^ The First Affiliated Hospital of University of Science and Technology of China (USTC), Division of Life Sciences and Medicine, University of Science and Technology of China, Hefei, China; ^2^ Department of Gynecology, Harbin Medical University Cancer Hospital, Harbin, China; ^3^ Department of Gynecology, Bengbu Medical College Bengbu, Anhui, China; ^4^ The First Affiliated Hospital of University of Science and Technology of China (USTC), Division of Life Sciences and Medicine, University of Science and Technology of China, Anhui Provincial Cancer Hospital, Hefei, China

**Keywords:** nicotinamide *N*-methyltransferase (NNMT), tumor biomarker, epigenetics, NNMT inhibitor, cancer therapy, tumor microenvironment

## Abstract

Cancer cells typically exhibit a tightly regulated program of metabolic plasticity and epigenetic remodeling to meet the demand of uncontrolled cell proliferation. The metabolic–epigenetic axis has recently become an increasingly hot topic in carcinogenesis and offers new avenues for innovative and personalized cancer treatment strategies. Nicotinamide *N*-methyltransferase (NNMT) is a metabolic enzyme involved in controlling methylation potential, impacting DNA and histone epigenetic modification. *NNMT* overexpression has been described in various solid cancer tissues and even body fluids, including serum, urine, and saliva. Furthermore, accumulating evidence has shown that *NNMT* knockdown significantly decreases tumorigenesis and chemoresistance capacity. Most importantly, the natural NNMT inhibitor yuanhuadine can reverse epidermal growth factor receptor tyrosine kinase inhibitor resistance in lung cancer cells. In this review, we evaluate the possibility of NNMT as a diagnostic biomarker and molecular target for effective anticancer treatment. We also reveal the exact mechanisms of how NNMT affects epigenetics and the development of more potent and selective inhibitors.

## Introduction

Cancer is one of the leading causes of death worldwide. GLOBOCAN 2020 has described the most recent changes in cancer burden and the importance of global cancer prevention and care (1). As many cancers lack specific symptoms, many patients are already in the advanced stage when the cancer is detected. Therefore, biomarkers that can be used for early diagnosis and detecting the therapeutic response are extremely important. Recently, the development of immunotherapy and targeted therapy has greatly improved patient prognosis, but acquired drug resistance needs to be addressed (2). Accordingly, more importance has been attached to cancer biological research, which aids the discovery of novel biomarkers, defining more effective molecular targets, and developing new treatment strategies.

Nicotinamide *N*-methyltransferase (NNMT) was identified as a cytoplasmic enzyme involved in methylating nicotinamide (NAM) and other structurally related compounds such as pyridines. NNMT coverts NAM into 1-MNA (1-methylnicotinamide), which is oxidized to N1-methyl-2-pyridone-5-carboxamide (2PY) and N1-methyl-4-pyridone-3-carboxamide (4PY) by AOX1 and eventually eliminated in the urine (3). However, recent findings have redefined NNMT function in physiology and disease. NNMT-mediated NAM and S-adenosyl methionine (SAM) depletion contributes to metabolic and epigenetic reprogramming by influencing epigenetic modifications and participating in different signaling pathways. This has yielded a broader understanding of various diseases, including metabolic disorder (4, 5), cancer (6–8), neurodegenerative diseases (9), and even endothelial disorder (10).

NNMT increases energy expenditure in a cell-autonomous manner by regulating histone methylation, polyamine flux, and SIRT protein, which suggests that NNMT is a potential target of diet-induced obesity and type 2 diabetes (11). NNMT is overexpressed in the stroma and body fluids and is associated with key prognostic parameters. It could be a novel, sensitive, and specific biomarker for aiding cancer detection (12, 13). Small interfering RNA (siRNA) knockdown of NNMT decreases cancer cell proliferative, migration, invasive, and resistance ability (14, 15). NNMT impairs methylation potential (MP) and decreases DNA and histone methylation, contributing to a wide range of changes in cancer-associated gene expression. Moreover, NNMT has been implicated in regulating autophagy to promote cancer cell survival and maintain cancer stem cell (CSC) stemness (15, 16). In addition to SAM depletion, NNMT enhances esophageal squamous cell chemoresistance by regulating the Warburg effect (6). Based on this, NNMT is a potential therapeutic target in several diseases. However, investigating its exact mechanism and developing efficient NNMT inhibitors remain research challenges.

This review summarizes the diagnostic value of NNMT in various tumors. We also focus on the potential mechanisms of NNMT promotion of cancer progression. Lastly, we introduce recent findings on NNMT inhibitors and their application in cancer therapy. This review provides an effective strategy for clinical transformation, which is a powerful weapon for treatment of patients with cancer.

## *NNMT* Expression and Gene Structure

NNMT is a cytosolic enzyme that was first purified from rat liver in 1951 (17). Subsequently, the *NNMT* gene was cloned and characterized by PCR, which contributed to revealing the possible molecular genetic mechanism of NNMT regulation (18). It was found that the *NNMT* gene is located on human chromosome 11q23.1 and mouse chromosome 9, and contains three exons and two introns (19, 20). The NNMT protein is relatively well-conserved across mammals, with 85% amino acid identity between humans and mice (3). The human *NNMT* gene is ~55.5-kb long and contains two major transcription initiation sites (TISs): TIS201 and TIS203. The product translated from TIS201 and TIS203 is also termed NNMT and consists of 265 amino acid residues. Analysis of the 2000 bp of genomic DNA upstream of TIS201 and TIS203 revealed no TATA box in either 5′ flanking sequence. Nevertheless, there are numerous potential STAT-binding elements, which are likely to regulate STAT3 responsiveness (21). STAT3 activation can improve *NNMT* expression and promoter activity (22). Hepatocyte nuclear factor 1β (HNF-1β) and interleukin-6 (IL-6) can regulate NNMT levels (22, 23). Combining NNMT and these regulators is likely to improve cancer prognosis. Single-nucleotide polymorphisms (SNPs) in and near the *NNMT* gene are also associated with many diseases. It is reported that there are 12033 variants across *NNMT* gene (24). A further illustration of this diversity in the genome comes from the Catalogue of Somatic Mutations in Cancer which lists 101 somatic mutations across the coding sections of NNMT, as a consequence changing or deleting 85 of the predominant amino acid residues (25). Association of SNPs within or near NNMT with cancer and non-cancerous illness, including neurologic illness, liver diseases, heart diseases, spina bifida and obesity has been described in detail. Skin cancers are most associated with somatic mutations across the *NNMT* coding sections. Furthermore, the NNMT SNP rs694539 increases risk of acute lymphoblastic leukemia (21).

NNMT is expressed mainly in the liver and adipose tissue, and a small amount of NNMT can be detected in the kidney, lung, skeletal muscle, and the heart (26). *NNMT* expression is altered in metabolic diseases such as obesity and cirrhosis (11). In addition, a wide range of studies have shown that NNMT is elevated in various cancers, including squamous cell carcinoma (SCC) (27), colorectal cancer (12), renal cell carcinoma (28), bladder cancer (29), ovarian cancer (30), cutaneous malignant melanoma (CMM) (31), gastric cancer (32), pancreatic cancer (33, 34), and glioblastoma (GBM) (7). Interestingly, NNMT expression was elevated in atorvastatin- and pravastatin-treated endothelial cells, which elevated nitric oxide and reactive oxygen species (ROS) (35). NNMT expression is usually elevated under metabolic stress and altered mitochondrial activity (36, 37), which may represent a strategy for reacting to oxidative stress.

## Association Between NNMT Expression and Poor Cancer Prognosis

Considering that NNMT is elevated in various cancers, most studies on NNMT have focused on its early diagnostic role (27, 38, 39). It is worth noting that some experiments have revealed increased NNMT expression in body fluids, including blood, urine, and saliva (12, 13, 40). NNMT is expected to be a novel serum tumor marker of lung cancer and colorectal cancer (40). Subsequently, Sartini et al. found that patients with oral SCC have higher salivary NNMT protein levels and activity than people without cancer, suggesting that NNMT could be a novel, specific, and low-invasive marker that aids cancer detection (41). It has even been proposed that NNMT be considered as a novel and sensitive serum biomarker for detecting colorectal cancer, outperforming the clinical significance of carcinoembryonic antigen (CEA) (42). Patients with gastric cancer with elevated CEA and NNMT had significantly poorer prognosis than those with only one positive marker, suggesting that combining NNMT with other biomarkers improves clinical diagnostic accuracy (43). It has also been suggested that NNMT expression was significantly higher in the urine of patients with bladder cancer compared with that detected in control specimens (44). Receiver operating characteristic analysis and area under the curve evaluation have demonstrated that NNMT has high sensitivity and specificity (13). Taken together, NNMT is expected to be a novel biomarker that can be applied to early and noninvasive diagnosis and prognosis. Nevertheless, NNMT is actually located in the cytoplasm, and the mechanism by which it is released to body fluids remains unclear.

In addition to being used in early diagnosis, NNMT is also related to poor prognosis. Elevated NNMT levels are typically associated with unfavorable clinical parameters such as tumor size and advanced stage and grade (27, 42). Moreover, *NNMT* gene silence promotes proliferation, migration, and therapy resistance. We summary association of NNMT expression with clinicopathological characteristics, the result of NNMT gene silence in patients with various cancers and the related signal transduction system (**Table 1**). Patients with high NNMT levels tend to have poor prognostic parameters and overall survival (OS) and disease-free survival (DFS) (26, 27, 45). We also list number of cases and hazard ratio of NNMT expression in the multivariate analyses. In pancreatic cancer, patients with tumor diameter > 4 cm (p < 0.001) and tumor-node-metastasis (TNM) stage III or IV (p < 0.001) have significantly higher NNMT levels than patients who do not (45). NNMT expression is also associated with CA19-9 level (P=0.005) but it is unclear whether the combination of NNMT and CA19-9 can improve clinical diagnostic accuracy in pancreatic cancers. In addition, NNMT expression in stomach adenocarcinoma correlates positively with the immune infiltration levels of monocytes, M2 macrophages, resting dendritic cells, and neutrophils, but correlates negatively with B cells (32). The NNMT expression score of high-grade serous carcinomas is higher than that in serous borderline tumors and low-grade serous carcinomas (38). Moreover, *NNMT* gene silence can increase radiosensitivity and chemosensitivity in GBM and CMM, which contributes to combating drug resistance and optimizing combination therapy. Nevertheless, Sartini et al. reported no connection between NNMT activity and non–small cell lung cancer (NSCLC) histologic subtype, grading, and pathological stage (61). It was confirmed that *NNMT* knockdown contributes to significant suppression of cell proliferation and anchorage-independent cell growth *in vitro* and *in vivo* (61). In addition, NNMT expression is not related to grade and stage in renal clear cell carcinoma but correlates negatively with tumor size, suggesting that it may play an important role in the initial malignant conversion phase (49). Unexpectedly, NNMT levels are decreased in human insulinoma and hepatocellular carcinoma (HCC), but the NNMT reduction mechanism remains unclear. In HCC, high *NNMT* mRNA levels tend to be connected with worse prognosis (26).

**Table 1:** Summary of the clinical value of NNMT in tumors.

Tumor	Clinical parameters	Effect of *NNMT* gene silencing	Value	Signal transduction systems	Case numbers	Hazard ratio(95% CI)	Reference(s)
Pancreatic cancer	Age, tumor size, TNM stage, poor differentiation, carbohydrate antigen (CA)19-9 level	Inhibits cell proliferation, invasion, and migration	Early diagnosis, prognostic indicator, distinguishing benign from malignant	–	178	0.399(0.284–0.560)	(45, 46)
Gastric cancer	Primary tumor size, lymph node metastasis, distant metastasis, TNM stage, immune infiltration	Inhibits cell proliferation, invasion, and migration	Early diagnosis, prognostic indicator, predicting outcome and immunotherapy response	TTPAL/NNMT/PI3K/AKT	781	1.446(1.041-2.065)	(32, 43, 47, 48)
HCC	Vascular invasion, distant metastasis, serum hepatitis B virus levels, liver cirrhosis status	Enhances autophagy, promotes tumor growth, influences phosphorylation	Prognostic indicator, early diagnosis	NNMT/CD44v3 NNMT/PP2A/ULK1	120	1.91(0.98-3.71)	(16, 26, 39)
Ovarian cancer	Histological subtype, advanced stage	–	Early diagnosis and prognostic indicator, reflects the degree of malignant and metastatic tumor behavior	BRCA1/NNMT/CDK1	103	2.5 (1.0–6.0)	(8, 38)
Renal cell carcinoma	Age, pT status, histology, tumor size	Inhibits cell invasion	Early and noninvasive diagnosis prognostic indicator	PI3K/AKT/SP1/MMP-2	74	–	(49–51)
Bladder cancer	Histological grade	Reduces cell migration	Noninvasive diagnosis, prognostic indicator	–	55	–	(52, 53)
Colorectal cancer	TNM stage, lymph node metastasis, differentiation grade (rectal cancer)	Suppresses cancer cell invasive capacity	Early and noninvasive diagnosis, prognostic indicator, chemotherapy resistance	NNMT/ASK1/p38/MAPK PI3K–Akt	1088	1.415 (1.015-1.972)	(12, 54–57)
SCC	Pathological stage, lymph node metastasis	Inhibits proliferation and tumorigenicity	Prognostic indicator, noninvasive diagnosis (oral SCC)	–	123	–	(27, 58, 59)
GBM	–	Increases sensitivity to radiation treatment	Prognostic indicator in combination with radiation treatment	NNMT/PP2A/STKs	–	–	(7, 37)
CMM	Breslow thickness, Clark level, presence/number of mitoses, ulceration	Reduces cell proliferation and migration, increases chemosensitivity to dacarbazine	Prognostic indicator, increases chemosensitivity	–	68	–	(31, 60)

Previous studies have mainly focused on NNMT upregulation and downregulation in whole tumor tissue. In fact, NNMT levels vary between the stroma and cancer cells and patients with high stromal NNMT tend to have a poor prognosis (38, 43, 62). Compared with other pathological types, the mesenchymal molecular subtype of ovarian cancer tends to have higher NNMT expression. NNMT expression, which can be used for decisions on prognosis and predicting bevacizumab treatment sensitivity (63). Moreover, high stromal NNMT expression is an independent risk factor of gastric cancer (62). Based on these findings, NNMT overexpression in the stroma aids cancer prognosis prediction.

## Biological Roles of the NNMT

Cancer cells and other cells in the tumor microenvironment (TME) have tumor heterogeneity and plasticity, which endows cancer cells with great adaptability to therapy, leading to drug resistance (64). Influenced by pro-tumorigenic cytokines, cancer cells acquire stem cell properties that promote cancer aggressiveness and resistance to therapy. Due to intra- and inter-tumoral heterogeneity, NNMT plays different roles in various cancers and cells. Herein, we review the potential mechanism by which NNMT promotes cancer progression and resistance.

### Cancer Cells

**Figure 1:**
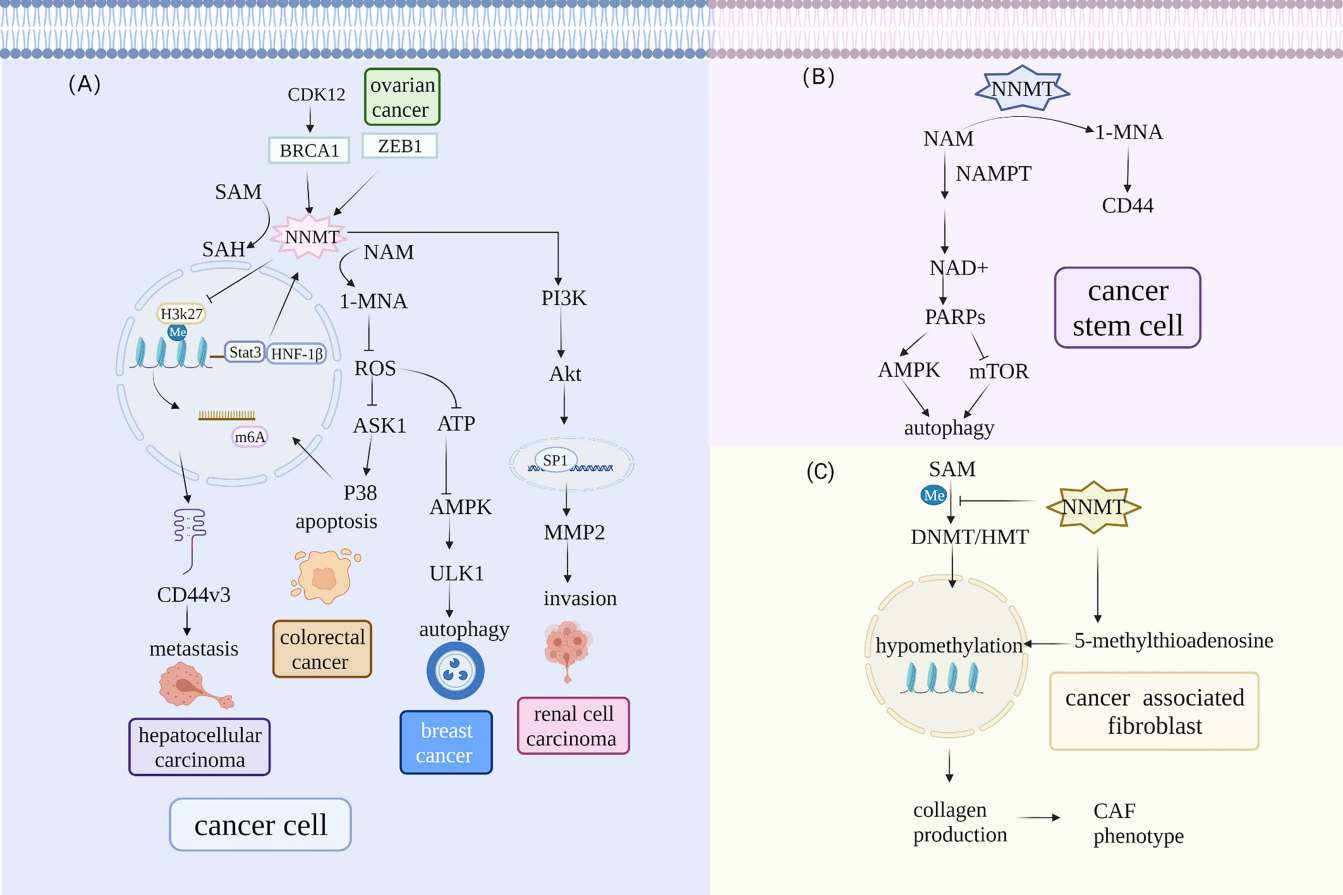
NNMT participates in different signaling pathways in the TME. **(A)** NNMT expression is directly regulated by transcription factors and subsequently affects metastasis, invasion, apoptosis, and autophagy *via* a series of downstream signal pathways in cancer cells. Hepatocyte nuclear factor 1β (HNF-1β) and STAT3 can regulate NNMT levels. Moreover, BRCA1 can occupy the *NNMT* promoter and regulate NNMT expression. In hepatic stellate cells, upregulated NNMT expression alters H3K27 methylation status and affects the N6-methyladenosine (m6A) of CD44, which contributes to tumor metastasis. NNMT promotes clear cell renal cell carcinoma invasion and metastasis by altering matrix metalloproteinase 2 expression. NNMT also lowers the phosphorylation of AKS1 that is an upstream signal of the p38-MAPK pathway and reduces apoptosis in colorectal cancer cells. NNMT can alter protein phosphatase2A methylation status and influence ULK1 activity to regulate autophagy in breast cancer. **(B)** NNMT regulates autophagy in CSCs by influencing NAD^+^ metabolism. NNMT decreases the activity of NAD+-dependent enzyme PARP1 that can influence autophagy by upregulation AMPK pathway and downregulation mTOR pathway in CSCs. **(C)** *NNMT* overexpression contributes to global hypomethylation and promotes CAF formation. NNMT can elevate 5-methylthioadenosine levels and contribute to global hypomethylation, which alters the methylation status of genes involved in collagen production and promotes Normal fibroblasts acquiring CAF phenotype.

NNMT is implicated in regulating tumor proliferation, invasion, and drug resistance by affecting multiple signaling pathways *in vivo* (**Figure 1**). The possible mechanism is that NNMT participates in regulating cell cycle progression *via* many pathways, such as the p27 and PI3K–Akt pathways, enhancing tumorigenesis capacity. *NNMT* silencing in MGC803 cells has demonstrated a significantly elevated percentage of G2-phase cells (43). On the contrary, downregulating *NNMT* decreased the proportion of G2- and S-phase cells. Although the results differ, they both suggest that NNMT increases tumorigenesis capacity by regulating the cell cycle. Xie et al. found that 1-MNA can decrease ROS levels, alter the NAD^+^/NADH ratio, and elevate intracellular ATP levels, suggesting that NNMT maintains cancer cell growth by affecting energy metabolism and promoting cell cycle progression (54). Others have demonstrated that 1-MNA can elevate complex I activity by reducing the degradation of NDUFS3, a complex I subunit. NNMT also can protect complex I from the toxicity of inhibitors such as 1-methyl-4-phenylpyridinium ion and rotenone (65). Lastly, NNMT overexpression unregulated CDK2 and CDK6 expression and downregulated P15, P21, and P27 expression, contributing to the imbalance between CDK and CKI. In this regard, NNMT promotes cancer cell proliferation *via* the p53–p21 and p27 pathways. Crucially, NNMT is also associated with the phosphorylation status of kinases in the PI3K–Akt and MAPK–ERK pathways (54). Upregulation of NNMT drives the metabolic alteration and sensitizes ovarian cancer cells to mitochondrial metabolic targeting agents without reducing proliferation. It was shown that BRCA1 occupied the *NNMT* promoter and regulating its expression (8). Specifically, *BRCA1* and *CDK12* depletion contribute to mitochondrial respiration defects and decreased ATP levels due to the reduced mitochondrial DNA copy number rather than mitochondrial number, which suggest that they stem from decreased flux through electron transport complexes rather than a decrease in mitochondria (8, 66). Furthermore, decreased ATP levels caused by BRCA1 depletion are driven primarily by upregulation of NNMT. Therefore, NNMT is expected to act as an effective cancer therapeutic target in *BRCA1*-deficient ovary tumor (8).

NNMT is also involved in regulating autophagy to maintain cancer cell survival *via* the mTOR and AMPK pathways. Autophagy is a highly conserved process with a crucial role in cancer status and CSC maintenance by yielding recycled metabolites for growth (67). *NNMT* knockdown protected hepatic cancer cells from nutrient starvation by promoting autophagy. Mechanistically, NNMT depletion reduced the consumption of methyl donors, increasing protein phosphatase2A (PP2A) methylation. Subsequently, PP2A decreased ULK1 activity through phosphorylated (p)-ULK1 (S638) dephosphorylation (16). ULK1 is an autophagy-related factor that can be regulated negatively by mTOR and positively by AMPK (68). 1-MNA decreases ROS levels, which can regulate autophagy *via* many signaling pathways (69) (70). Furthermore, a recent study determined that NNMT and 1-MNA regulated autophagy through the ROS-mediated AMPK pathway, protecting breast cancer cells from oxidative stress (71). It is still not clear which signaling pathways NNMT is involved in for regulating autophagy. Nevertheless, these results suggest that NNMT-mediated autophagy may be a new target therapeutic intervention for human cancer.

More importantly, NNMT plays an important role in chemoresistance by participating in the PI3K–Akt and p38–MAPK pathways (15, 72). In NSCLC cell lines with gefitinib resistance, including H292, PC9, H1993 and HCC827 cell lines, *NNMT* expression is inversely related to that of miR-449a. NNMT stimulates gef-resistant NSCLC cell growth by targeting miR-449a. Knockdown of *NNMT* (0.5μM) enhanced sensitiveness of H292-Gef cells compared with their control (7.6μM). Mechanistically, NNMT can be upregulated by PI3K–Akt pathway, which is negatively regulated by PTEN. Knockdown of *NNMT* significantly increased microRNA (miR)-449a levels and decreased the levels of active Akt *in vivo* in that miR-449a reduced PTEN methylation. This positive feedback loop appears to be a key factor in NNMT overexpression and chemoresistance. Combining NNMT inhibitors and miR-449a enhances antitumor capacity *in vivo* and *in vitro* (73). Recently, *NNMT* overexpression has been found to stabilize SIRT1 protein and reduce the chemotherapy efficacy of adriamycin and paclitaxel in breast cancer cells in an obvious manner. Although *SIRT1* mRNA was not significantly changed, SIRT1 protein expression was elevated (15). NNMT regulated glucose, lipid, and cholesterol metabolism in the liver by affecting *SIRT1* expression and stability. The results suggest that NNMT and 1-MNA reduce proteasome degradation of SIRT1 by reducing SIRT1 ubiquitination (4). Likewise, a further study suggested that NNMT enhances chemoresistance to 5-FU (5-fluorouracil) in colorectal cancer cells. The possible mechanism is that NNMT decreases 5-FU-induced ROS production in colorectal cancer cells, which lowers the phosphorylation of ASK1, an upstream signal of the p38–MAPK pathway (55). Therefore, NNMT can predict chemotherapy efficacy and represents a potential antitumor target. Nevertheless, the exact mechanisms of NNMT have not been fully investigated and more studies are required to elucidate the molecular mechanism.

### Stromal Cells

Previous studies have indicated that NNMT expression is elevated in the stroma of many cancers (56, 74). Immunohistochemistry-based analysis of NNMT in gastric cancer showed that it is mainly expressed in stromal cells (62). Stromal cells play an important role in tumor heterogeneity and promote tumor cell plasticity *via* metabolic and epigenetic signals. Understanding the reason for the high NNMT expression and its role in stromal cells may contribute to elucidating the mechanism of tumor progression. Hepatic stellate cells had upregulated NNMT expression and altered H3K27 methylation status, which affected the N6-methyladenosine (m6A) of CD44, consequently contributing to the formation of the splice variant CD44v3, which is closely related to tumor metastasis. Meanwhile, 1-MNA reduced the ubiquitin-mediated degradation of CD44 and stabilized CD44 protein, promoting cancer cell invasion and migration (39). Accordingly, NNMT involvement in stromal cell and cancer cell interaction in the mechanical microenvironment may be a reason for the tumor metastasis. A subsequent study has indicated that NNMT promotes clear cell renal cell carcinoma invasion and metastasis by inducing matrix metalloproteinase 2 (*MMP2*) expression. NNMT alters Akt phosphorylation and affects the association between the transcription factor SP1 and the *MMP2* promoter (50). *NNMT* knockdown in a more aggressive squamous cell line contributed to the downregulation of epithelial–mesenchymal transition (EMT)-related genes, including *MMP9*, osteopontin (*OPN*), and versican core protein (*VCAN*), which are involved in focal adhesion and extracellular matrix (ECM)–receptor interaction (14). Although preliminary, these findings indicate that NNMT is also involved in regulating EMT. Nonetheless, the association between NNMT-induced EMT and tumor invasion and the detailed signaling pathways involved in this process are unclear.

Cancer-associated fibroblasts (CAFs) are a fibroblast subpopulation that secrete pro-oncogenic cytokines and ECM-modifying factors promoting and supporting malignant cell growth and invasion (75). Other cells, such as epithelial cells, can transdifferentiate to CAFs *via* MMPs, ROS, and tumor-derived transforming growth factor-β (TGF-β) (76). A recent proteomics-based study has revealed that NNMT is a main metabolic regulator of CAFs in ovary cancer (74). *NNMT* mRNA expression is associated positively with CAF markers (56). In CAFs, NNMT can elevate 5-methylthioadenosine levels, contributing to global hypomethylation. This alters the methylation status of genes involved in collagen production, enhancing collagen contractility (74). Normal fibroblasts acquire the CAF phenotype and secrete many cytokines and oncogenic ECM that contribute to tumor growth and metastasis. Furthermore, *NNMT* knockdown can convert CAFs to normal fibroblasts, which suggests bidirectional interconversion (74). These results indicate that NNMT drives CAF oncogenic behavior and is also a novel treatment target that can normalize the stroma and attenuate cancer cell invasion.

### Cancer Stem Cells

Tumor initiation and progression is driven by CSCs that have the ability to self-renew and differentiate into cells to avoid clone depletion (77). Due to their role in escaping immune surveillance and chemotherapy/apoptosis, more attention should be focused on the potential mechanism. NNMT is elevated in CSCs (7, 78). For example, glioma stem cells (GSCs) have significantly higher NNMT expression than matched differentiated tumor cells. Compared with proneural tumors, NNMT is preferentially expressed in mesenchymal GSCs, a more aggressive subtype. siRNA inhibition of *NNMT* reduces GSC growth and self-renewal *in vitro* and is more effective than nicotinamide phosphoribosyltransferase (NAMPT). The findings highlight the possibility of identifying NNMT as a specific GSC phenotype and propose that NNMT is involved in maintaining GSC stemness (7). Regulating methylation can directly lead to metabolic conversion in cancer cells with different phenotypes and even enable cancer cell acquisition of stem cell properties (79). In mesenchymal GSCs, *NNMT* expression contributes to DNA methylation by participating in nicotinate and methyl donor metabolism (7). As mentioned above, NNMT increases the production of a CD44 variant, a surface marker widely used for identifying CSCs (39). Concordantly, glucose deprivation, which robustly induced *NNMT* expression in the OVCAR3 cell line, increased the proportion of CD44-expressing cancer stem-like cells. NNMT is elevated with expression of ZEB1, a specific CSC transcription factor, but not all NNMT-positive cells are associated with *ZEB1* overexpression. Therefore, NNMT-positive cells may acquire stem cell properties *via* a ZEB1-independent mechanism (80). The acquisition of such stem cell properties may have a detrimental effect on cancer invasion and drug resistance. Recently, it has been shown that NNMT is likely to regulate autophagy to maintain CSC stemness. Specifically, NNMT can decrease the activity of the PARP1, which is an enzyme to synthesize poly (ADP-ribose) using NAD^+^ as a substrate (81). PARP1 can influence autophagy by upregulating the AMPK pathway and downregulating the mTOR pathway (67). Such evidence strongly indicates that NNMT might represent a novel antitumor target based on CSC-associated targets.

### NNMT Facilitates Energy Metabolism Alterations to Maintain Cancer Cell Survival

Reprogramming energy metabolism is an emerging hallmark of tumor (82). NNMT is a positive regulator of gluconeogenesis and its knockdown in adipose tissue prevented diet-induced obesity in mice (4, 11). Both glucose-6-phosphatase catalytic (G6pc) (20%) and phosphoenolpyruvate carboxykinase 1 cytosolic (Pck1) (40%) expression are significantly decreased in primary hepatocytes with *NNMT* knockdown. Further studies showed that NNMT can regulate Sirt1 expression and the stability of Sirt1 protein by lowering Sirt1 ubiquitination (4). It has been reported that Sirt1 is a regulator of gluconeogenic/glycolytic pathways (83, 84). The effect of NNMT on gluconeogenesis is mediated by Sirt1 (4). Warburg effect is a phenomenon that even in the presence of oxygen, cancer cells rely on the glycolytic pathway and produce lactate (85). NNMT is a key regulatory element of cancer cell metabolism, especially the Warburg effect. In esophageal squamous cell carcinoma lines, gas chromatography–mass spectrometry analysis has shown that TE1 cells with low sensitivity to 5-FU have higher glycolysis activity and NNMT levels than EC1 and EC109 cells. *NNMT* knockdown significantly decreased glucose consumption and the expression of glycolysis-related enzymes, including HK2, LDHA, and PAGM1, thereby increasing the 5-FU sensitivity of EC1 cells (6). Silencing *PGAM1* upregulated 3-phosphoglycerate (3-PG) and 2-PG levels, inhibiting aerobic glycolysis (86). Moreover, the glycolytic inhibitor 2-deoxyglucose (2‐DG) reversed 5-FU sensitivity in NNMT overexpression cells. Consistent with this, NNMT knockdown increased 2-DG resistance in PANC-1 cells. Recent evidence suggests that in ovarian cancer, NNMT expression is associated positively with PGAM1, which is involved in the Warburg effect, enabling the elucidation of the resistance mechanism of bevacizumab. NNMT is expected to be used for predicting ovarian cancer sensitivity to bevacizumab. Combining an NNMT inhibitor and bevacizumab contributes to improving survival (63). These findings show that NNMT is involved in regulating the Warburg effect enhancement of chemoresistance and is considered a candidate target for cancer therapy. Instead of an energy generation pathway, the Warburg effect presents a mode of energy conversion, generating biosynthetic intermediates to yield precursors for other pathways (87). The exact mechanisms of NNMT regulation of the Warburg effect are not fully understood.

Owing to high NNMT expression, glucose-restricted ovarian cancer cells can acquire metabolic adaptations, using other sugars and some methylated substrates to feed their metabolism. It is possible that NNMT alters histone and DNA methylation status, inducing epigenetic remodeling and metabolism reprogramming (88). Moreover, whether it is in glucose-dependent or glucose-independent ovarian cancer cells, ectopic ZEB1 can increase the expression of *NNMT* and EMT-associated genes, including *MMP2*, *CTGF*, and *SPARC*. In fact, EMT is a result of phenotypic and metabolic plasticity induced by the ZEB1–NNMT axis (80). NNMT directly increases CTGF expression by reducing *CTGF* promoter methylation (89). This is a new pathway connecting metabolism and mesenchymal gene expression and contributing to explanation of the mechanisms of cancer cell survival and metastasis in nutrient deficiency.

## The Crosstalk Between Tumor Metabolism and Epigenetics

Accumulating evidence has shown that, in cancer, cellular metabolism can also have a profound impact on signaling and transcriptional networks *via* different mechanisms (87). It is widely accepted that cancer cells reprogram the nutrient procurement and metabolism pathways to meet the demands of uncontrolled cell proliferation (85). Moreover, many intermediary metabolites generated from glycolytic and oxidative phosphorylation serve as cofactors or substrates of epigenetic enzymes involved in post-translational modification and gene expression. Therapies targeting the epigenome have been involved in the potential reinvigoration of antitumor immunity.

### NNMT Regulates NAD^+^ Metabolism to Influence Deacetylases

**Figure 2:**
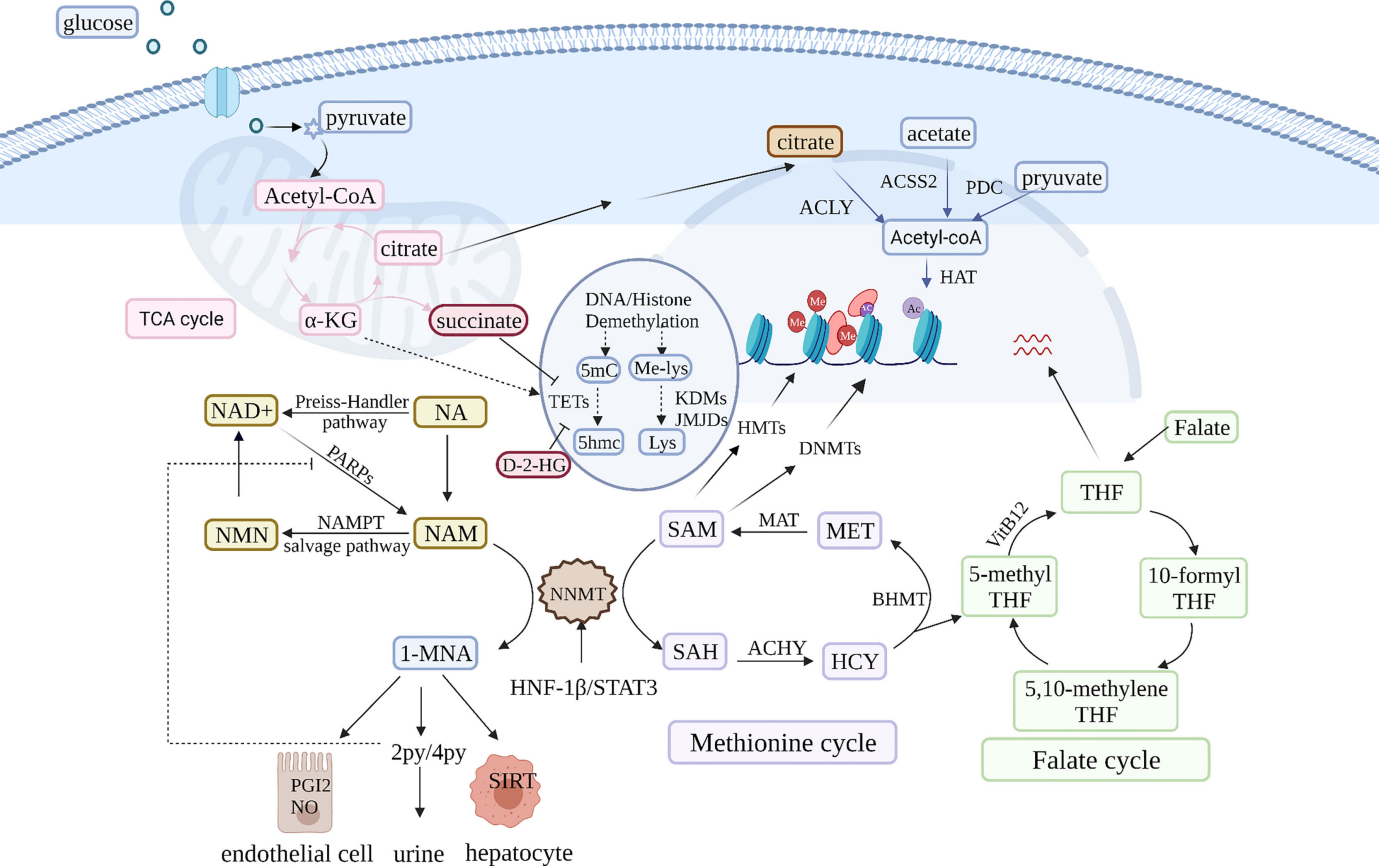
NNMT is pivotal in NAM and SAM metabolism. NNMT uses the universal methyl donor SAM and converts NAM into 1-MNA, which oxidized to 2PY and 4PYand eventually eliminated in the urine. NNMT overexpression leads to a dual drain of NAM and SAM in cancer cells. On one hand, it affects the regeneration of NAD^+^, a coenzyme of various glucose metabolism enzymes. NNMT over expression decreases NAD^+^ and contributes to glucose metabolism disorders. The glucose metabolism intermediate α-ketoglutarate (α-KG) is a cofactor of the ten-eleven translocation methylcytosine hydroxylases (TET) and Jumonji C demethylases family (JMJC HDM), which are involved in DNA and histone demethylation, respectively. Furthermore, some glucose metabolism products can enter the nucleus and affect epigenetics. On the other hand, deleting the methyl donor SAM contributes to global hypomethylation and affects the expression of a variety of cancer-associated genes. NNMT also can regulate methyl donor balance by interacting with some enzyme in methionine cycle. In addition to NAM and SAM metabolism, 1-MNA can directly increase Sirt1 protein expression independent of its mRNA level. Sirt1, a regulator of gluconeogenesis and cholesterol synthesis is closely related to NAD^+^ metabolism. Therefore, NNMT is a pivot in multiple metabolism cycles and influence cancer metabolism from many aspects.

In addition to metabolism remodeling, epigenomic reprogramming is another main consequence of NNMT overexpression. Chromatin status is driven by the activity and level of chromatin-modifying enzymes such as DNA methyltransferases (DNMT) and histone deacetylases (HDAC) (90) (**Figure 2**). These enzymes are involved in methylation and acetylation and depend on metabolites as cofactors, integrating the epigenome and transcriptome. NAD^+^ metabolism is a basis of epigenetic regulatory metabolic reprogramming (91). Under physiological conditions, NAD^+^ is converted to NAM in NAD^+^-consuming reactions catalyzed by sirtuins and PARPs (92). NAD^+^ is required for maintaining the activity of the sirtuin class of HDACs, which NAM inhibits (90). NNMT is activated under NAM saturation conditions because NNMT has low affinity for NAM (3). NNMT prevents NAM accumulation and its inhibitory effect on NAD^+^-dependent enzymes. Nevertheless, NNMT overexpression is likely to disrupt the balance between NAD^+^ synthesis and breakdown and decrease NAD^+^-dependent enzyme activity (79). These enzymes are sensitive to metabolite concentrations and their activity directly influences epigenetic modifications (90). NAM increases SIRT1 activity *via* NNMT and 1-MNA (4). Moreover, NAM-treated NNMT overexpression mice exhibit fatty liver deterioration because NNMT decreases NAD^+^ levels and SIRT3 activity, downregulating the expression of genes involved in fatty acid oxidation (5). Therefore, it is important to investigate the influence of NAM concentrations on NNMT-regulated chromatin modification in cancer cells.

### Disruption of Methyl Donor Balance in NNMT Overexpression Cancer Cells

NNMT is also a key node in methyl donor metabolism. NNMT uses the universal methyl donor SAM and produces increased concentrations of 1-MNA, which is further oxidized to 2-PY and 4-PY and eventually excreted in the urine (90). Based on findings that cancer cells have limited capacity for recovering methylation units, Ulanovskaya et al. proposed that 1-MNA is a methylation sink (88). NNMT-mediated SAM depletion can alter cell MP, contributing to the change in global epigenetic histone profiles in cancer (7, 93). For example, NNMT can alter H2K27 methylation status, affecting m6A of CD44, consequently contributing to the formation of CD44v3, which is closely related to tumor metastasis (39). Recent preliminary findings have demonstrated that triple-negative breast cancer (TNBC) cell lines had significantly higher NNMT levels than estrogen receptor-positive cell lines. NNMT overexpression in TNBC cells reduced m6A modification in an obvious manner, contributing to the oncogenic phenotype (94). In addition, *NNMT* knockdown upregulated tumor suppressor PP2A activity in GBM by increasing methyl availability for leucine carboxyl methyl transferase 1 (LCMT1) and increasing PP2A methylation while concomitantly decreasing the activity of serine/threonine kinases such as Akt and p38 MAPK (95). This aids the understanding of how NNMT affects signaling pathways in cancer cells to promote tumor proliferation, metastasis, and resistance.

Generally, NNMT overexpression decreases SAM and S-adenosyl-L-homocysteine (SAH) levels and alters SAM/SAH *in vivo* and *in vitro* in an obvious manner (96). Nevertheless, NNMT knockdown had no effect on SAM/SAH and NAD^+^ levels in the liver (4). On the contrary, SAM/SAH was significantly decreased in NAM-supplemented NNMT overexpression mice. The possible reason is that NNMT is not a major methyltransferase in the liver. Under the condition of absence of glycine *N*-methyltransferase (GNMT), NNMT exhibited obvious regulation of MP in normal tissues (5, 88). Multiple factors affect NNMT regulation in hepatic methyl donor balance, which may be related to NNMT downregulation in HCC. In addition to SAM depletion, even inactive NNMT can interact with betaine-homocysteine methyltransferase (BHMT), methionine adenosyltransferase (MAT), and adenosylhomocysteinase (AHCY) to regulate methyl donor balance (96, 97). NNMT promotion of DNA hypomethylation relies on methionine concentrations, a metabolic regulator of methyl donors (7). NNMT over expressing cancer cells that have grown in 20 μM methionine had a more dramatic reduction of SAM levels than those that have grown in 100 μM methionine (88). Furthermore, methionine restriction upregulated NNMT expression and increased mesenchymal gene expression by reducing promoter region methylation. NNMT and methionine restriction both reduced DNMT1 and DNMT3A expression, which may be another DNA hypomethylation mechanism (7).

## Development and Application of NNMT in Cancer Therapy

Epigenetic and transcriptomic reprogramming during cancer development is increasingly important and is expected to be an attractive target for cancer treatment. Given the therapeutic hypotheses associated with NNMT, selective, potent, and cell-active inhibitors would be a valuable tool for assessing its therapeutic potential. There are three main NNMT inhibitor types: 1) competitive; 2) bisubstrate; and 3) covalent, with different activities and characteristics (**Table 2**).

**Table 2:** The present NNMT inhibitors.

Type	Name	IC_50_	Characteristics	Reference(s)
Competitive inhibitors	SAH	35.3 ± 5.5 μm	Competes with SAM	(47)
	1-MNA	24.6 ± 3.2 μm	Competes with NAM; used in biochemical activity assays	(98)
	Sinefungin	17.0 ± 3.4 μm	A broad-spectrum methyltransferase inhibitor; SAM-dependent	(98)
	5-Amino-1MQ	1.2 ± 0.1 μm	A low-micromolar inhibition substrate mimetic inhibitor	(99, 100)
	JBSNF-000088	0.588 ± 0.075 μm	A small-molecule inhibitor; regulates MNA levels	(19)
	Compound 2	1.6 μm	A tricyclic compound	(101)
Bisubstrate inhibitors	Compound 78	1.41 μm	Incorporates a naphthalene moiety; has a dose-dependent inhibitory effect on cancer cell proliferation	(47)
	MS2734	14 ± 1.5 μm	Noncompetitive with the NAM substrate and competitive with SAM; high selectivity	(102)
	Compound 45	29.2 ± 4.0 μm	A trivalent compound; the best mimic of the NNMT transition state	(98)
	LL320	1.6 ± 0.3 nm (K_i_)	A reversible, tight-binding inhibitor; the first propargyl-linked bisubstrate analog; poor cell permeability	(103)
	NS1	0.5 nm (K_i_)	The most potent NNMT inhibitor	(104)
Covalent inhibitors	RS004	10 μm	Targets the Cys165 residue in the SAM-binding pocket	(105)
	HS312	0.35/0.18 μm	Inhibitory effect correlates negatively with SAM concentrations; engages other protein targets *in situ*	(106)
Natural product	YD	0.4 μm	Combines with NNMT interaction and overcomes resistance in NSCLC	(73)

Byproducts of NNMT, SAH and 1-MNA inhibit NNMT activity *via* a feedback mechanism (107). A study of comprehensive structure–activity relationships (SARs) revealed that quinolinium analogues inhibit NNMT at low concentrations and selectively bind to the NNMT substrate-binding site residues (99). In diet-induced obesity mice, 5-amino-1MQ (5-amino-1-methylquinolinium) exhibited high membrane permeability and caused weight loss by suppressing lipogenesis (100). Based on the primary findings for NNMT in obesity and type 2 diabetes, a recent experiment demonstrated that JBSNF-000088-treated high-fat diet-induced obesity mice exhibited weight loss, improved insulin sensitivity, and normal glucose tolerance (19). However, JBSNF-000088 had no effect on *Nnmt* knockout mice, suggesting that it is specific to some extent (19). The above findings show that NNMT inhibitors are effective in metabolic disorders, but the potential corrective roles between metabolism and epigenetics in cancer cells are not fully understood. Moreover, bisubstrate inhibitors are designed to target both the substrate and cofactor binding sites to enhance activity and selectivity. This characteristic may contribute to mimicking the ternary transition state of a bireactant enzymatic reaction (102). However, their poor cell permeability has limited their use. SAHH (*S*-adenosylhomocysteine hydrolase)-coupled assay showed that MS2734 inhibited NNMT at a median inhibitory concentration (IC50) of 14 ± 1.5 μm (102). Presently, NS1 is the most potent and selective NNMT inhibitor in **Table 2**. Inhibiting alkynyl bisubstrate was a generalizable strategy that is expected to be used for developing probes and inhibitors of other methyltransferases. A naphthalene moiety increased the activity of a bisubstrate inhibitor of NNMT, which was supported by isothermal titration calorimetry binding assays and modeling studies (47). An alpha-chloroacetamide (αCA) compound, RS004 is the first reported covalent inhibitor and inhibits NNMT by covalently binding the noncatalytic active site Cys165 residue (105). The natural product yuanhuadine (YD) significantly inhibits NNMT expression and upregulates miR-449a levels in EGFR-TKI-resistant NSCLC in a concentration-dependent manner. Furthermore, the structure of the NNMT-YD complex had four hydrogen bonds and that YD suppresses NNMT activity *via* the interacting pocket of the enzyme. These findings aid the development of novel NNMT inhibitors. YD enhances sensitivity to EGFR-TKIs and combining NNMT inhibitors and other traditional drugs improves patient prognosis (73). In addition to the three inhibitor types, *N*-methyl-4-chloropyridine irreversibly inhibits NNMT based on suicide inhibition, where NNMT promotes its methylation and it subsequently reacts with C159 to inhibit NNMT (108). Currently, it is unclear whether strong or weak NNMT inhibition benefits humans. Experimental studies on the role of NNMT inhibitors in humans are still needed.

Given the urgent demand for NNMT inhibitors, there is a need for a convenient and efficient assay to assess NNMT therapeutic effects. Directly determining the binding affinities of inhibitors using isothermal titration calorimetry and surface plasmon resonance is too costly. SAH-derived chemical proteomic probes and SAH photoreactive probes for profiling NNMT activity have been reported (105, 106). Iyamu et al. designed and synthesized a II138 fluorescent probe (FP) and established an FP-based competition assay to directly detect the molecules that interact with the NNMT active site (109). The authors suggested that this economical and robust assay is appropriate for high-throughput screening applications. However, the FP assay could not detect suicide inhibitors that do not need to bind SAM (109). In short, better tools are needed for identifying and quantifying NNMT inhibitor activity.

At present, many noncancer drugs have shown antitumor effects, including cardiovascular drugs, antibiotics, and psychotropic drugs (110, 111). Drug repurposing is increasingly popular because it lowers cost, has sufficient pharmacological data, and ensures clinical experiment security. Statins affected hepatoma cell migration, invasiveness, and CD44 expression and decreased NNMT levels obviously and in a dose-dependent manner (39). However, a key problem of drug repurposing is the lack of single-agent activity, its feasibility should be examined in further studies (112).

## Discussion

As we have summarized, preliminary studies have shown that NNMT plays a key role in various cancers and is expected to be a novel therapeutic antitumor target. In tumor microenvironment, NNMT promotes cancer progress and chemoresistance by regulating cell cycle and autophagy in cancer cells, stromal cells and even cancer stem cells. NNMT in metabolic/epigenetic axis reveals potential mechanism of cancer progress. NNMT can regulate NAD^+^ Metabolism and disrupt methyl donor balance in cancer cells. Despite the progress in understanding NNMT functions, knowledge gaps remain. Further experiments are required to identify the signals regulating NNMT expression and clarify the exact mechanism and pathways involving NNMT. NNMT and 1-MNA promote tumor progression *via* various signaling pathways. Nevertheless, it is unclear that whether 2-PY and 4-PY are active and affect the TME *via* other cell communications, including autocrine or paracrine. The conserved function of NNMT is best reflected by identifying the changes it causes in multiple cancer cell types. Nonetheless, NNMT has different roles in obesity, type 2 diabetes, and hepatic disease (4, 5). Revealing the potential mechanisms is important.

Due to heterogeneity, NNMT metabolic and epigenetic regulation vary between different cancers and specific TME regions. It is unknown whether NNMT is involved in maintaining methyl donor balance in specific diseases and individuals. Specifically, 1-MNA regulates the immune system in ovarian cancers (113). In addition, NNMT expression in stomach adenocarcinoma correlates positively with the immune infiltration levels of monocytes, M2 macrophages, resting dendritic cells, and neutrophils, but correlates negatively with B cells (32). Accordingly, the authors suggested that NNMT can be used for guiding immunotherapy (32). The present review does not cover the role of NNMT in the immune microenvironment. NNMT functions in a specific cell population can be analyzed using single-cell RNA sequencing (scRNA-seq) (114). Organoids are a three-dimensional culture system *in vivo* that can simulate tissue and organ physical functions (115, 116). Combining organoids and scRNA-seq can identify TMEs and cellular heterogeneity in greater detail, aiding the definition of NNMT functions in different diseases and the development of advanced personalized treatment for patients with metastatic and chemoresistant cancers.

Furthermore, due to the lack of specific and efficient NNMT inhibitors, it remains uncertain whether NNMT can be a novel drug target. NNMT inhibitor development faces challenges. The first crystal structure of human NNMT in a complex with a small-molecular inhibitor formed a solid structural basis for NNMT inhibitor development (102). High selectivity can be achieved by targeting a small binding pocket, such as the NAM-binding pocket. One direction is enhancing bisubstrate inhibitor cellular potency. Inhibitors with high selectivity and efficacy contribute to elucidating the potential mechanism of NNMT and developing novel therapeutic strategies. Combining NNMT inhibitors and traditional targeted drugs, including EGFR-TKIs and bevacizumab, can improve prognosis (63, 73). Therefore, NNMT inhibitors can also be used to combat drug resistance and optimize combination therapy.

We expect that NNMT is a novel biomarker that can be used for early and noninvasive diagnosis. Furthermore, it plays an important role in tumor progression by regulating methyl donor metabolism and participating in multiple signaling pathways. With the development of more studies and clinical trials, we strongly believe that NNMT inhibitors can confer considerable benefit on patients with cancer.

## Author Contributions

X-YL collected the related papers and was a major contribution in writing the manuscript. Y-NP made figures and tables. YC and QZ revised the article. B-RX initiated the study and revised the manuscript. All authors read and approved the final manuscript.

## Funding

This study was supported by the National Natural Science Foundation of China (No. 81872430), Special Fund in China Postdoctoral Science Foundation (No. 2019T120281, 2019M661304), Heilongjiang Province Postdoctoral Science Foundation (No. LBH-Z18109) and Natural Science Foundation of Heilongjiang Province (No. H2017049) to B-RX.

## Conflict of Interest

The authors declare that the research was conducted in the absence of any commercial or financial relationships that could be construed as a potential conflict of interest.

## Publisher’s Note

All claims expressed in this article are solely those of the authors and do not necessarily represent those of their affiliated organizations, or those of the publisher, the editors and the reviewers. Any product that may be evaluated in this article, or claim that may be made by its manufacturer, is not guaranteed or endorsed by the publisher.

## Abbreviations

NNMT, nicotinamide *N*-methyltransferase; NAM, nicotinamide; 1-MNA, 1-methylnicotinamide; 2-py, N1-methyl-2-pyridone-5-carboxamide; 4-py, N1-methyl-4-pyridone-3-carboxamide (4py); AOX, aldehyde oxidase; SAM, S-Adenosyl methionine; SAH, S-adenosyl-L-homocysteine; MP, methylation potential; CSC, cancer stem cell; TISs, transcription initiation sites; HNF-1β, hepatocyte nuclear factor 1β; IL-6, interleukin-6; SNPs, single-nucleotide polymorphisms; TME, the tumor microenvironment; EGFR-TKI, epidermal growth factor receptor tyrosine kinase; m6A, N6-methyladenosine; *MMP2*, matrix metalloproteinase 2; NSCLC, non–small cell lung cancer; NAMPT, nicotinamide phosphoribosyltransferase; FP, fluorescent probe; YD, yuanhuadine; GNMT, glycine *N*-methyltransferase; DNMT, DNA methyltransferases; HDAC, histone deacetylases; BHMT, betaine-homocysteine methyltransferase; MAT, methionine adenosyltransferase; 5-amino-1MQ, 5-amino-1-methylquinolinium; HCC, hepatocellular carcinoma; SCC, squamous cell carcinoma; GBM, glioblastoma; CMM, cutaneous malignant melanoma; Ki, inhibition constant.
